# Terpene Research Is Providing New Inspiration for Scientists

**DOI:** 10.3390/molecules26185480

**Published:** 2021-09-09

**Authors:** Pavel B. Drasar, Vladimir A. Khripach

**Affiliations:** 1 Department of Chemistry of Natural Compounds, University of Chemistry and Technology, Technicka 5, 166 28 Prague, Czech Republic; 2 Institute of Bioorganic Chemistry, National Academy of Sciences of Belarus, 5/2 Academician V. F. Kuprevich Street, BY-220141 Minsk, Belarus; khripach@iboch.by

This current Special Issue of *Molecules* gathers selected communications on terpenes and terpene derivatives, clearly demonstrating the sustained interest in and importance of natural products in this field; fields connected to secondary metabolites; and renewable resources of plant and animal compounds for medicinal, material, supramolecular, and general chemistry research.

This issue gathers 17 papers, 5 review articles, and 12 research communications with a vast range of topics with different perspectives, such as the optimization of FRAP methodology for studies based on selected monoterpenes [1], and the improved functioning of fibroblasts and endothelial cells and accelerated healing of cutaneous wounds in mice caused by uvaol [2]. It presents a new iridoid and three new bis-iridoid glycosides from the leaves of *Lasianthus verticillatus*, lasianosides F–I [3], and atypical lindenane-type sesquiterpenes from *Lindera myrrha* [4]. It comprises a study of antibacterial and antifungal sesquiterpenoids from the aerial parts of *Anvillea garcinii* [5] and phytochemical and safety evaluations of volatile terpenoids from *Zingiber cassumunar* on mature carp peripheral blood mononuclear cells and embryonic zebrafish [6]. Similarly, it presents the cytotoxic and anti-inflammatory effects of *ent*-kaurane derivatives isolated from the alpine plant *Sideritis hyssopifolia* [7]. The issue also lists the prenyleudesmanes and a hexanorlanostane from the roots of *Lonicera macranthoides* [8]. There is discussion on the quantitative analysis of terpenic compounds in microsamples of resins by capillary liquid chromatography [9] and high-throughput ^1^H-NMR-based screening for the identification and quantification of heartwood diterpenic acids in four black pine *Pinus nigra* marginal provenances in Greece [10]. This Special Issue presents the naturally occurring pentacyclic triterpene betulin, which promotes differentiation of human osteoblasts in vitro and exerts an osteoinductive effect on the hFOB 1.19 cell line through activation of JNK, ERK1/2, and mTOR kinases [11]. Two new 11,20-epoxybriaranes are described from the gorgonian coral *Junceella fragilis* from Ellisellidae [12].

The review articles describe the issue of topical administration of terpenes encapsulated in nanostructured lipid-based systems [13]; an overview of the synthesis, modification, and biological activity of diosgenyl β-d-glycosaminosides [14]; metabolic engineering of *Escherichia coli* for the production of lycopene [15]; traps and pitfalls—unspecific reactions in metabolic engineering of sesquiterpenoid pathways [16]; and the recent achievements in medicinal and supramolecular chemistry of betulinic acid and its derivatives [17].

The Special Issue also aims to commemorate the great work and legacy of Prof. Kenji Mori, providing important material not only to many chemists but also to the specialists in several other fields.
